# Impact of birth weight on adult-onset diabetes mellitus in relation to current body mass index: The Japan Nurses' Health Study

**DOI:** 10.1016/j.je.2016.08.016

**Published:** 2017-06-20

**Authors:** Kota Katanoda, Mitsuhiko Noda, Atsushi Goto, Hideki Mizunuma, Jung Su Lee, Kunihiko Hayashi

**Affiliations:** a Division of Cancer Statistics Integration, Center for Cancer Control and Information Services, National Cancer Center, Tokyo, Japan; b Department of Endocrinology and Diabetes, Saitama Medical University, Saitama, Japan; c Department of Diabetes Research, Diabetes Research Center, National Center for Global Health and Medicine, Tokyo, Japan; d Epidemiology and Prevention Group, Center for Public Health Sciences, National Cancer Center, Tokyo, Japan; e Fukushima Medical Center for Children and Women, Fukushima Medical University, Fukushima, Japan; f Department of Public Health, Graduate School of Medicine, The University of Tokyo, Tokyo, Japan; g Department of Basic Allied Medicine, School of Health Science, Gunma University, Gunma, Japan

**Keywords:** Birth weight, Body mass index, Diabetes mellitus, Type 2, Fetal development, Small for gestational age

## Abstract

**Background:**

Although birth weight is considered as a fetal determinant of the development of adult-onset diabetes mellitus (DM), its public health importance relative to adult body mass index (BMI) remains unclear. We aimed to examine the association between adult-onset DM and birth weight in relation to adult BMI.

**Methods:**

We conducted a self-administered questionnaire as a baseline survey of the Japanese Nurses' Health Study cohort between 2001 and 2007. Exclusion criteria were applied to the volunteer sample of 49,927 female nurses (age <30 years or unknown, current pregnancy, development of DM before the age of 30 years, unknown core variables), and data from 26,949 female nurses aged 30 years or older were used. The association between history of DM diagnosis and birth weight was analyzed using logistic regression.

**Results:**

A linear inverse association was observed between birth weight and DM, after adjustment for age, BMI, and parental history of DM. The odds ratio for developing DM per 100 g increase in birth weight was 0.93 (95% confidence interval [CI], 0.90–0.96). The association was unchanged when birth weight was converted to percentile for gestational age. In the BMI-stratified analysis, the odds ratio for DM in the <2500 g birth weight group reached 4.75 (95% CI, 1.22–18.44, compared to the reference 3000–3499 g group) among women with normal low BMI (18.5–20.9).

**Conclusions:**

Birth weight and its percentile for gestational age were associated with adult-onset DM. Attention should be paid to the risk of DM among women born with low weight, even when their current BMI is normal.

## Highlights

• Association of birth weight and adult-onset DM was analyzed using JNHS study data.
• Odds ratio for developing DM per 100 g increase in birth weight was 0.93.
• The link was also observed when birth weight percentile for gestational age was used.
• Low birth weight was associated with adult-onset DM, even with normal current BMI.

## Introduction

Adult-onset diabetes mellitus (DM) is a major global medical burden. Globally, an estimated 1.3 million deaths were attributed to DM in 2010, twice as many as in 1990.^1^ There have been systematic reviews of the association between birth weight and adult-onset DM,^2^, ^3^ one of which reported that the risk of DM decreased by 30% for every 1 kg increase in birth weight.^3^ Although the finding provided support for the theory of fetal origins of adult disease,^4^ the impact of birth weight in relation to adult body mass index (BMI) is still unknown. Obesity in adulthood is a major risk factor for DM. The effect of birth weight on adult-onset DM is reported to vary depending on BMI in adulthood.^5^ According to the above-mentioned systematic review, the association between birth weight and DM is weaker in people with a higher current BMI.^3^ However, important questions have not been addressed, such as whether we should be cautious about developing DM even when current BMI is normal, and how DM risk changes according to the combination of birth weight and current BMI levels.^5^

The association between birth weight and adult-onset DM is biologically explained as an adaptation to intrauterine undernutrition.^4^ If this hypothesis is true, the effect of being small for gestational age will be more important. Although the association between birth weight and DM has been reported in many studies, few studies have investigated the effect of fetal growth on the development of DM.^6^

In Japan, the prevalence of low birth weight has been increasing. According to the national statistics from 2012, the proportions of infants with a birth weight of less than 2500 g were 8.5% in males and 10.7% in females. These proportions were almost double those from 1980 (4.8% in males and 5.6% in females).^7^ Estimation of the impact of birth weight on later life is needed to evaluate the public health burden of the increasing prevalence of low birth weight.

The present study aimed to examine the association between adult-onset DM and birth weight using data from a cohort of Japanese female nurses. To clarify the meaning of the association in the practice of public health and obstetrics, current BMI categories were used as adjustment, stratification, or combined independent variables, and the effect of birth weight for gestational age was examined using percentile score based on fetal growth curve.

## Materials and methods

### Data

The baseline data from the cohort of the Japanese Nurses' Health Study (JNHS) were used. JNHS is an ongoing prospective cohort study in female nurses that was started in 2001.^8^ The baseline survey, which used a self-administered questionnaire, was conducted from 2001 to 2007, and responses were obtained from 49,927 female nurses. The participants of the present study were female nurses aged 30 years or older. After the following exclusion criteria were applied, the analytic cohort included 26,949 women: age <30 years or unknown (2179 women), current pregnancy (944 women), unknown DM status (53 women), development of DM before the age of 30 years (37 women), unknown birth weight (19,328 women), and unknown current BMI (437 women).

### Variables

The variables used in the present study were history of adult-onset DM diagnosis, birth weight (four categories: <2500 g, 2500–2999 g, 3000–3499 g, and ≥3500 g), current BMI (six categories: <18.5, 18.5–20.9, 21.0–22.9, 23.0–24.9, 25.0–26.9, and ≥27.0 kg/m^2^), and maternal or paternal history of DM.

History of a DM diagnosis was determined via the question “Have you ever been diagnosed with diabetes by a physician?” For this question, gestational diabetes was explicitly excluded. As stated above, we excluded participants diagnosed with diabetes before age 30 and classified the remaining cases as adult-onset DM cases.

Percentiles of birth weight for gestational age were calculated based on the Japanese neonatal anthropometric charts.^9^ Specifically, from the birth weight and gestational age at birth of each participant, a z-score of birth weight was determined using the LMS method proposed by Cole.^10^ The z-scores were converted into percentile scores on the assumption of a normal distribution. Percentile scores were used because they are widely used in obstetric practice to evaluate intrauterine nutritional status. Smoking status (three categories: current smoker, former smoker, and nonsmoker) was also used in stratified analysis. Women with unknown gestational age (8701 women) and those with an extremely high percentile score (>99.9%) or an extremely low percentile score (<0.1%) (703 women) were excluded from the statistical analyses that included the percentile score. We applied this exclusion criterion because several unrealistic combinations of gestational age and birth weight were found in both extreme ends. We confirmed in a preliminary analysis including all data that this exclusion did not affect our main result.

### Statistical analysis

Logistic regression analysis was performed with adult-onset DM status as the dependent variable and birth weight as the independent variable. An age-adjusted model, an age- and current BMI-adjusted model (model 1), and a model additionally adjusted for maternal/paternal history of DM (model 2) were applied. Stratified analyses were performed according to BMI, presence or absence of maternal/paternal history of DM, and smoking status. The 3000–3499 g birth weight category was used as the reference. Because there were few women in the BMI category of <18.5 kg/m^2^ and the category of former smokers, these categories were excluded from the stratified analyses on the association between birth weight and adult-onset DM. For the analysis of birth weight percentile, BMI categories with small numbers of participants were rounded (18.5–21.9 and 22.0–24.9 kg/m^2^ for stratified analysis according to BMI; <21.0 kg/m^2^ for other stratified analysis). To examine the effect of large birth weight in detail, an additional analysis was done in which the highest birth weight group was divided into two categories (3500–3999 g and ≥4000 g).

In order to compare the effects between birth weight and BMI, a model using a combination of birth weight and current BMI as the independent variable was applied. The participants were classified into a total of 16 categories according to the combination of the four birth weight categories and the four BMI categories (18.5–20.9, 21.0–22.9, 23.0–24.9, and ≥25.0 kg/m^2^). The combined category of BMI of 18.5–20.9 kg/m^2^ and birth weight of 3000–3499 g was used as a reference to calculate odds ratios because the category was most frequent within the normal or ordinary range.

Logistic regression analysis was also performed with birth weight percentile as the independent variable and adult-onset DM status as the dependent variable. Birth weight percentiles were divided into five categories: <10th, 10th–29th, 30th–69th (reference), 70th–89th, and ≥90th.

In order to check the validity of the outcome variable, sensitivity analysis was performed using a dependent variable of DM defined by a combination of data, including a fasting plasma glucose level of ≥126 mg/dL and the use of DM drugs, obtained from the baseline questionnaire survey. Analysis including only women with DM diagnosed within the previous 3 years was also done.

Ethical approval for the study was obtained from the Ethics Review Committees of the Faculty of Medicine, Gunma University and the National Institute of Public Health.

## Results

Table 1 shows the characteristics of the participants according to the birth weight categories. The number of women was largest in the 3000–3499 g birth weight group (43.0%) and smallest in the <2500 g group (8.8%). Mean age decreased as birth weight increased (linear trend, *p* < 0.001). The mean gestational age increased as birth weight increased (linear trend, *p* < 0.001). There was a U-shaped association between current BMI and birth weight (quadratic trend, *p* < 0.001), and the 2500–2999 g birth weight group had the lowest current BMI. Current smokers accounted for approximately 18% of the participants, and the number of current smokers tended to be larger in groups with a higher birth weight (linear trend, *p* = 0.03). The proportion of women with adult-onset DM ranged from 0.6% to 1.6%, and was smaller in the groups with a higher birth weight (linear trend, *p* < 0.001). The number of women with a paternal history of DM tended to be smaller in the groups with a higher birth weight, whereas the number of women with a maternal history of DM tended to be larger in the groups with a higher birth weight (linear trend *p* < 0.001 and *p* < 0.001, respectively). Women with a maternal history of DM accounted for 9.5% and 12.9% of those with a birth weight of 3500–3999 g and ≥4000 g group, respectively, whereas the percentages were 7%–8% for those with a birth weight less than 3500 g.

**Table 1:** Baseline characteristics according to birth weight categories.

	Birth weight				
	<2500 g	2500–2999 g	3000–3499 g	≥3500 g	All
Number of participants	2371 (8.8%)	8967 (33.3%)	11,585 (43.0%)	4026 (14.9%)	26,949 (100.0%)
Age, years, mean (SD)	40.1 (7.2)	39.6 (6.9)	39.3 (6.9)	38.3 (6.7)	39.3 (6.9)*
Age, years					
30–39 years	53.6%	55.9%	57.0%	64.2%	57.4%
40–49 years	34.1%	33.4%	33.5%	28.2%	32.8%
50–59 years	11.8%	10.3%	8.9%	7.1%	9.3%
≥60 years	0.5%	0.4%	0.5%	0.6%	0.5%
Gestational age, weeks,^a^ mean (SD)	36.3 (5.7)	37.6 (6.9)	38.2 (6.8)	38.7 (6.2)	37.9 (6.7)*
<32 weeks	5.0%	1.4%	1.1%	0.6%	1.4%
32–36 weeks	33.8%	5.1%	1.4%	1.4%	5.5%
37–41 weeks	58.6%	90.0%	92.7%	88.4%	88.2%
42+ weeks	2.6%	3.6%	4.8%	9.6%	4.9%
Current BMI, kg/m^2^, mean (SD)	21.8 (3.2)	21.5 (3.0)	21.7 (3.0)	21.9 (3.2)	21.7 (3.1)*
<18.5	11.8%	11.3%	9.9%	8.5%	10.4%
18.5–20.9	36.3%	39.7%	37.8%	37.5%	38.3%
21.0–22.9	23.8%	24.5%	25.5%	26.3%	25.2%
23.0–24.9	14.9%	13.1%	14.2%	13.7%	13.8%
25.0–26.9	6.4%	6.5%	6.6%	7.2%	6.6%
≥27.0	6.8%	4.8%	6.0%	6.8%	5.8%
Smoking status					
Never	69.4%	70.6%	69.5%	68.0%	69.7%
Current	17.9%	16.5%	18.0%	18.5%	17.6%*
Former	11.7%	11.7%	11.4%	12.5%	11.7%
(Unknown)	1.0%	1.2%	1.0%	0.9%	1.0%
History of adult-onset DM	1.6%	1.0%	0.7%	0.6%	0.8%*
Paternal history of DM	15.3%	13.7%	12.6%	12.1%	13.1%*
Maternal history of DM	8.6%	7.3%	7.8%	10.1%	8.1%*

Values reported as n (%), unless otherwise noted.

DM, diabetes mellitus.

* Significant difference across birth weight categories (*p* < 0.05).

a Participants with unknown gestational period were excluded (8701 women).

Table 2 shows the results of the analysis of the association between birth weight category and adult-onset DM status. In all three models (age-adjusted; age- and BMI-adjusted; and age-, BMI-, and parental DM-adjusted models), a linear inverse association was observed between birth weight and adult-onset DM. In the model adjusted for age, BMI, and parental DM history (model 2), the odds ratio for developing DM per 100 g increase in birth weight was 0.93 (95% confidence interval [CI], 0.90–0.96). Results were similar when the analysis was limited to full-term birth (odds ratio according to 100 g increase in birth weight, 0.92; 95% CI, 0.88–0.96). In the additional analysis, in which the highest birth weight group was divided into two categories (3500–3999 g and ≥4000 g), the linear decreasing trend did not change (odds ratio compared with the reference birth weight group, 1.12; 95% CI, 0.69–1.81 and 0.20; 95% CI, 0.03 to 1.45, respectively). The analysis according to the BMI categories revealed that, even among women with normal low BMI (18.5–20.9), the prevalence of DM was significantly higher in women with a lower birth weight (trend, *p* = 0.013), with a significant odds ratio of 4.75 (95% CI, 1.22–18.44) for the birth weight <2500 g compared with the reference birth weight group (3000–3499 g). Although without statistical significance, the BMI groups between 21.0 and 24.9 kg/m^2^ also showed a similar tendency (trend, *p* = 0.09). Among the women with a BMI of 27.0 kg/m^2^ or higher, the prevalence of DM was significantly higher in the <2500 g, 2500–2999 g, and ≥3500 g birth weight groups than the reference birth weight group, with point estimates of odds ratios exceeding 2.0. The trend indicating a lower prevalence of DM in those with a higher birth weight was also shown in analyses according to the presence or absence of paternal history of DM and smoking status. The analysis according to maternal history of DM revealed a significant association between birth weight and DM status only in women without a maternal history of DM (trend *p* = 0.873 and *p* < 0.001 for those with and without maternal history of DM, respectively). Interaction between parental history of DM and birth weight was significant for maternal but not paternal history of DM (*p* = 0.029 and *p* = 0.408, respectively). Regarding the factors other than body weight, the age-adjusted odds ratio of paternal history of DM, maternal history of DM, and current smoking was 3.48 (95% CI, 2.59–4.67), 3.73 (95% CI, 2.76–5.04), and 1.02 (95% CI, 0.71–1.46), respectively.

**Table 2:** Association between birth weight and diagnosis of adult-onset DM.

	Birth weight													
(Number of participants; %)	<2500 g (2371; 8.8%)			2500–2999 g (8967; 33.3%)			3000–3499 g (11,585; 43.0%)	≥3500 g (4026; 14.9%)			*P for Trend*^f^	100 g increase in birth weight		
	OR	95% CI		OR	95% CI		(reference)	OR	95% CI			OR	95% CI	
Age-adjusted	**2.20**	1.48	3.27	**1.52**	1.12	2.07	(1.00)	1.00	0.63	1.60	**<0.001**	**0.94**	0.91	0.97
Model 1 (age + BMI adjusted^a^)	**2.37**	1.59	3.54	**1.70**	1.24	2.33	(1.00)	0.93	0.58	1.49	**<0.001**	**0.93**	0.90	0.96
Model 2 (Model 1 + parental DM history adjusted)	**2.34**	1.56	3.52	**1.73**	1.26	2.37	(1.00)	0.94	0.58	1.50	**<0.001**	**0.93**	0.90	0.96
By BMI^b^, kg/m^2^														
18.5–20.9	**4.75**	1.22	18.44	2.05	0.59	7.13	(1.00)	0.61	0.06	6.01	**0.013**	**0.86**	0.78	0.95
21.0–22.9	1.56	0.60	4.03	1.16	0.58	2.31	(1.00)	0.40	0.09	1.76	0.083	0.94	0.88	1.01
23.0–24.9	1.74	0.71	4.25	1.08	0.53	2.21	(1.00)	0.54	0.16	1.87	0.089	0.95	0.89	1.02
25.0–26.9	**2.92**	1.10	7.70	1.76	0.86	3.59	(1.00)	0.48	0.13	1.72	**0.003**	**0.90**	0.84	0.96
≥27.0	**2.89**	1.40	5.98	**2.12**	1.16	3.86	(1.00)	**2.01**	1.01	3.98	0.179	**0.95**	0.90	1.00
By history of paternal DM^c^														
Yes	1.96	0.92	4.17	1.52	0.85	2.70	(1.00)	0.51	0.17	1.54	**0.009**	**0.91**	0.87	0.97
No	**2.62**	1.62	4.25	**1.80**	1.23	2.62	(1.00)	1.11	0.65	1.88	**<0.001**	**0.93**	0.90	0.96
By history of maternal DM^d^														
Yes	1.80	0.75	4.34	1.08	0.55	2.09	(1.00)	1.46	0.68	3.14	0.873	0.98	0.92	1.03
No	**2.63**	1.66	4.19	**1.93**	1.34	2.78	(1.00)	0.69	0.36	1.30	**<0.001**	**0.91**	0.88	0.94
By smoking status^e^														
Never smokers	**1.96**	1.20	3.21	**1.77**	1.24	2.53	(1.00)	0.90	0.52	1.57	**<0.001**	**0.93**	0.90	0.96
Current smokers	**3.13**	1.28	7.65	0.98	0.39	2.46	(1.00)	0.83	0.27	2.52	**0.020**	0.94	0.88	1.01

Bold font indicates statistical significance (*p* < 0.05).

CI, confidence interval; OR, odds ratio; DM, diabetes mellitus.

a BMI was classified into six categories (<18.5, 18.5–20.9, 21.0–22.9, 23.0–24.9, 25.0–26.9, 27.0+).

b Adjusted for age and parental DM history. The category of BMI<18.5 was not included because of limited number of participants.

c Adjusted for age (continuous), BMI (six categories above) and maternal DM history.

d Adjusted for age (continuous), BMI (six categories above), and paternal DM history.

e Participants with unknown smoking status were excluded (285 women). Adjusted for age (continuous), BMI (six categories), and parental DM history.

f Median birth weight of each category was entered into the model.

**Fig. 1:**
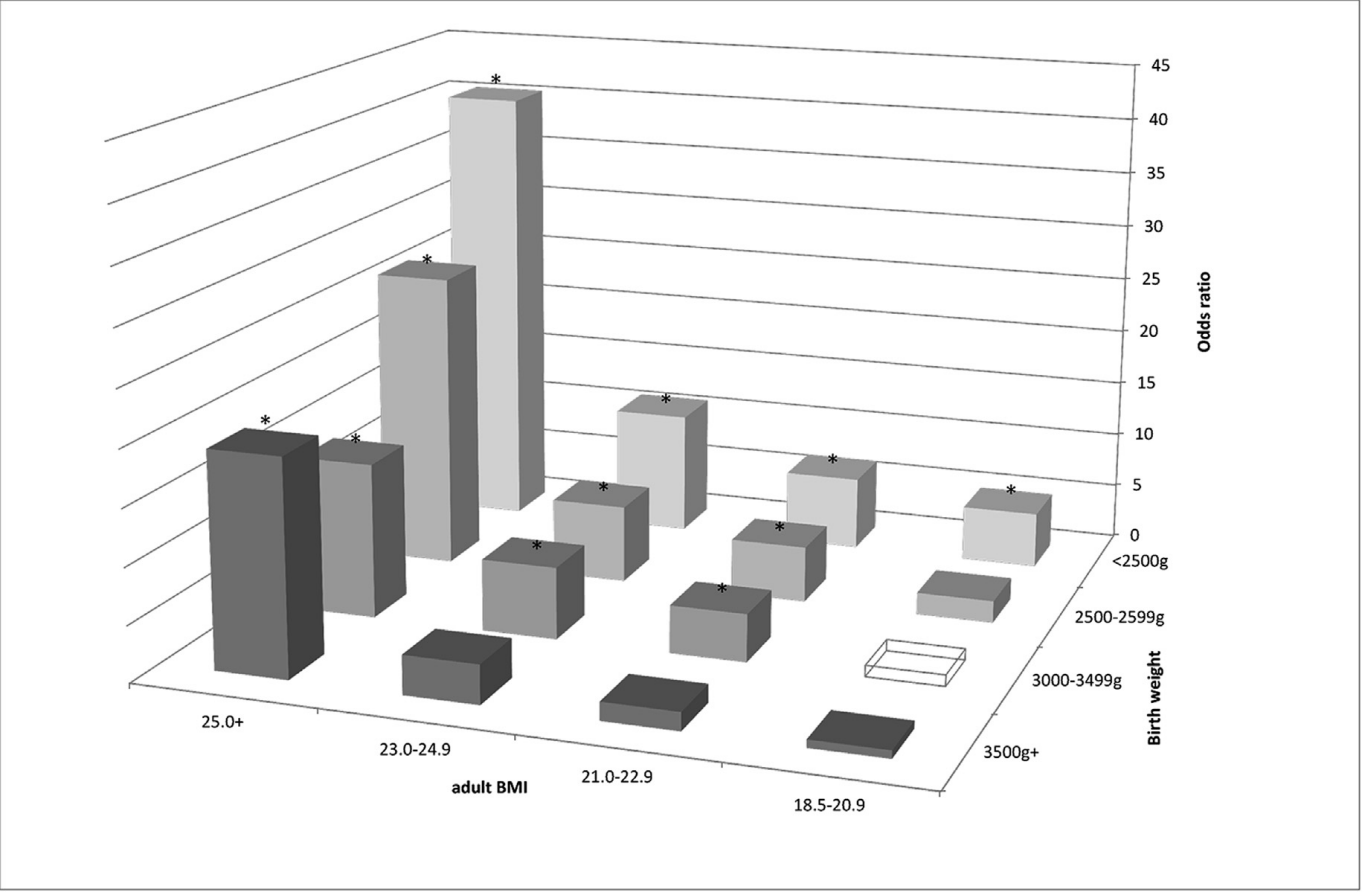
Odds ratio of adult-onset DM according to the combined categories of birth weight and adult BMI. Note: The reference group was a combination of a birth weight of 3000–3499 g and a BMI of 18.5–20.9 kg/m^2^, shown as a transparent box. * Statistically significant (*p* < 0.05).

Fig. 1 shows the results of the analysis of the association between adult-onset DM status and the combined categories of birth weight and adult BMI. Women with a BMI of 25.0 kg/m^2^ or higher had a significantly higher DM prevalence regardless of their birth weight, with an odds ratio exceeding 13.0 compared with the reference group with a birth weight of 3000–3499 g and a BMI of 18.5–20.9 kg/m^2^. The maximum odds ratio reached 40.4 (95% CI, 13.6–120.2) for those with a birth weight of <2500 g and a BMI of >25.0 kg/m^2^. Women born with a birth weight of less than 2500 g had significantly higher prevalence of DM than the reference group, regardless of their current BMI.

Table 3 shows the results of the analysis of the association between birth weight percentiles for gestational age and adult-onset DM status. The odds ratio for developing DM per 10-percentile increase in birth weight was 0.91 (95% CI, 0.86–0.96) (trend, *p* = 0.001). Compared to the group at the 30th–69th percentile of birth weight, the odds ratios for developing DM were 1.63 in the group at the 10th–29th percentile (95% CI, 1.06–2.50) and 2.59 in the group below the 10th percentile (95% CI, 1.59–4.21). This trend persisted in the analysis that included only women born at full term. The analysis on birth weight percentiles according to the BMI categories revealed that, even among women with normal BMI (22.0–24.9), the prevalence of DM was significantly higher in women with a lower birth weight percentile (trend, *p* = 0.043). Among women with a normal but small BMI (18.5–21.9), the prevalence of DM was significantly higher in the <10 percentile category, with an odds ratio 3.01 (95% CI, 1.12–8.08). The trend indicating a lower prevalence of DM in those with a higher birth weight percentile was also shown in analyses according to the presence or absence of paternal history of DM. Similar to the result of the analysis of birth weight categories per se, the analysis according to maternal history of DM revealed an association between birth weight percentile and DM status only in women without a maternal history of DM. Interaction between parental history of DM and birth weight percentile was significant for maternal but not paternal history of DM (*p* = 0.045 and *p* = 0.092, respectively). Regarding smoking status, a significant association between birth weight percentile and DM was observed in never smokers alone.

**Table 3:** Association between birth weight percentile and diagnosis of adult-onset DM.^a^, ^b^

	Birth weight percentile																
(Number of participants; %)	<10th percentile (1454; 8.2%)			10–29th percentile (2904; 16.4%)			30–69th percentile (6848; 38.8%)	70–89th percentile (3319; 18.8%)			≥90th percentile (3144; 17.8%)			*P for Trend*^g^	10 point increase in brith weight percentile		
	OR	95% CI		OR	95% CI		(reference)	OR	95% CI		OR	95% CI			OR	95% CI	
Gestational period																	
All^b^	**2.59**	1.59	4.21	**1.63**	1.06	2.50	(1.0)	1.39	0.87	2.23	0.96	0.57	1.61	**0.001**	**0.91**	0.86	0.96
Full term only (37=<42 weeks)^b^	**2.42**	1.44	4.07	**1.68**	1.08	2.61	(1.0)	1.20	0.72	2.00	0.88	0.50	1.54	**<0.001**	**0.90**	0.84	0.95
By BMI^c^, kg/m^2^																	
18.5–21.9	**3.01**	1.12	8.08	1.15	0.39	3.40	(1.0)	1.20	0.37	3.90	0.96	0.26	3.54	0.166	0.91	0.80	1.04
22.0–24.9	2.03	0.87	4.75	1.22	0.56	2.68	(1.0)	1.33	0.58	3.01	0.30	0.07	1.29	**0.043**	**0.90**	0.81	1.00
25.0–26.9	2.74	0.78	9.60	1.80	0.70	4.62	(1.0)	2.05	0.73	5.76	0.55	0.15	2.09	0.083	0.89	0.78	1.01
≥27.0	2.11	0.77	5.74	2.21	1.00	4.90	(1.0)	1.08	0.42	2.75	1.78	0.80	3.95	0.225	0.94	0.86	1.04
By history of paternal DM^d^																	
Yes	2.18	0.88	5.38	1.87	0.87	4.04	(1.0)	1.37	0.55	3.38	0.41	0.09	1.84	**0.017**	**0.87**	0.78	0.98
No	**2.85**	1.59	5.09	1.49	0.88	2.52	(1.0)	1.40	0.81	2.45	1.18	0.67	2.08	**0.029**	**0.93**	0.87	0.99
By history of maternal DM^e^																	
Yes	2.97	0.87	10.11	1.41	0.56	3.57	(1.0)	**2.77**	1.17	6.52	1.34	0.51	3.53	0.777	1.02	0.91	1.14
No	**2.61**	1.53	4.45	1.61	0.99	2.63	(1.0)	1.06	0.59	1.91	0.82	0.43	1.55	**<0.001**	**0.88**	0.83	0.94
By smoking status^f^																	
Never smokers	**2.78**	1.61	4.79	1.63	1.00	2.65	(1.0)	1.18	0.67	2.07	0.91	0.50	1.67	**<0.001**	**0.89**	0.84	0.95
Current smokers	2.37	0.64	8.80	1.67	0.47	5.90	(1.0)	2.76	0.94	8.10	0.93	0.25	3.40	0.617	0.97	0.85	1.10

Bold font indicates statistical significance (*p* < 0.05).

CI, confidence interval; OR, odds ratio; DM, diabetes mellitus.

a Birth weight percentile was calculated using the population average and standard deviation of birth weight according to gestational period. Participants with unknown gestational age (8701 women) or extreme birth weight percentile (703 women) were excluded.

b Adjusted for age (continuous), BMI (six categories: <18.5, 18.5–20.9, 21.0–22.9, 23.0–24.9, 25.0–26.9, 27.0+), parental DM history, and gestational period (continuous).

c Adjusted for age (continuous), parental DM history, and gestational period (continuous). BMI categories were rounded because of small numbers of participants.

d Adjusted for age (continuous), BMI (five categories: <21.0, 21.0–22.9, 23.0–24.9, 25.0–26.9, 27.0+), maternal DM history, and gestational period (continuous).

e Adjusted for age (continuous), BMI (five categories: <21.0, 21.0–22.9, 23.0–24.9, 25.0–26.9, 27.0+), paternal DM history, and gestational period (continuous).

f Adjusted for age (continuous), BMI (five categories: <21.0, 21.0–22.9, 23.0–24.9, 25.0–26.9, 27.0+), parental DM history, and gestational period (continuous).

g Birth weight percentile was entered into the model.

In the sensitivity analysis about the dependent variable of DM, using a combination of a fasting plasma glucose level of ≥126 mg/dL and the use of DM drugs revealed a similar inverse association between birth weight and DM (trend, *p* = 0.006, after adjustment of age, BMI, and parental DM history). Moreover, analysis that included only women with DM diagnosed within the previous 3 years revealed the same results (trend, *p* < 0.001).

Birth weight was unknown in approximately 40% of our study population. When sensitivity analysis was performed by including these women in either the lowest or highest birth weight category, there was no marked change in the inverse association with DM status. This result suggests that the effects of unknown birth weight on the major findings of the present study are negligible.

## Discussion

The present study, which used data from a cohort of Japanese female nurses, revealed a linear inverse association between birth weight and adult-onset DM status. The odds ratio of 0.93 (95% CI, 0.90–0.96) per 100 g increase in birth weight adjusted for age, current BMI, and parental history of DM corresponds to an odds ratio of 0.48 per 1 kg increase (95% CI, 0.36–0.64). This indicates a more substantial association than that found in the meta-analysis reported in 2010 (odds ratio 0.70; 95% CI, 0.65–0.76, adjusted for age, sex, and current BMI).^3^ Several Japanese studies also reported an inverse association between birth weight and DM.^11^, ^12^ The reported odds ratio in one study was larger than that observed in the present study (3.52 for birth weight <2500 g compared with 3001–3200 g), but with a wide confidence interval (95% CI, 1.04–11.96).^12^ The present study confirmed the association with a larger sample distributed throughout Japan.

The association between birth weight and adult-onset DM was different according to BMI in adulthood. Specifically, in overweight adults (BMI ≥25.0 kg/m^2^), the risk of DM is high regardless of birth weight, whereas women with a normal BMI tended to have a high risk of DM when they were born with a low birth weight or their birth weight was small for gestational age. In Japan, at present, obesity is defined as a BMI of ≥25.0 kg/m^2^, and preventive measures against lifestyle-related diseases, including DM, are implemented at this BMI level. The results of the present study show the importance of being aware of the increased risk of DM in adults with a lower birth weight even when their BMI is within a normal range.

Several population-based studies have reported a U-shaped association between birth weight and adult-onset DM.^2^, ^3^, ^13^, ^14^ These studies revealed an increased risk of DM in the birth weight categories of ≥4000 g. In our additional analysis, in which the highest birth weight group was divided into two categories (3500–3999 g and ≥4000 g), the linear decreasing trend did not change. Analysis stratified by birth weight percentile categories (small for gestational age, appropriate, or large for gestational age) and maternal or paternal history of DM also revealed the same tendency of linear inverse association, except in women with a maternal history of DM (eTable 1). Our result adds supporting evidence to the finding of the meta-analysis that most middle-aged and older populations in the later 20th century have inverse birth weight-DM associations.^3^ It should be noted that such linear inverse associations were not observed for obese women. For women with BMI ≥27.0, the trend was not significant, and the odds ratios were significantly high whether the birth weight was smaller or larger than the reference group (Table 2). This result was similar in the analysis using birth weight percentile (Table 3). The relation between birth weight and future DM may be modified by the existence of adult obesity.

High birth weight is associated with a maternal history of DM.^14^ In the present study, we also observed a higher percentage of women with a maternal history of DM among those with a heavier birth weight. The association between high birth weight and maternal history of DM may increase odds ratios for DM in people with a high birth weight. Our results are consistent with this possibility: the odds ratios for adult-onset DM in the groups with a birth weight of 3500 g or more were above 1.0 in the women with a maternal history of DM and below 1.0 in those without a maternal history of DM (Table 2). These opposite directions of association were also observed in the analysis using birth weight percentiles. Although our questionnaire did not distinguish pre-pregnancy DM and gestational DM for maternal history of DM, our result is consistent with the notion that U-shaped birth weight-DM associations are observed only in a population with high prevalence of obesity and DM.^3^, ^14^

The analysis using birth weight percentiles for gestational age also revealed a linear inverse association with adult-onset DM status. This result suggests that intrauterine fetal development or nutritional status affects the association between birth weight and DM. The result, showing that the risk of DM decreases by 10% for every 10-percentile increase in fetal weight, is useful evidence for the management of fetal weight in obstetric practice. This inverse linear association was similar across different BMI categories in adulthood, regardless of the history of paternal DM.

In the present study, the younger the participants were, the higher birth weights they tended to have (Table 1). This is because the majority of the study population was born before 1980, when an increasing trend in birth weight was observed in the entire Japanese population (female infants: 3060 g in 1960, 3140 g in 1980, and 2990 g in 2000).^15^ Thus, it is possible that birth weights acted as a surrogate of a cohort effect. However, an additional stratified analysis by the baseline age of 50 years did not alter the inverse association between birth weight and DM (trend, *p* < 0.001 in the <50-year-old group and *p* = 0.006 in the ≥50-year-old group). Given the fact that the prevalence of low birth weight has recently been increasing in Japan,^7^ we have an increasing proportion of children with a higher risk of adult-onset DM.

The strength of the present study is that the women were widely distributed throughout Japan and were healthcare workers.^8^ The results of the sensitivity analysis of DM status and the validity survey for self-reported birth weight confirm the accuracy of the responses to the questionnaire.

The limitations of the present study include the cross-sectional study design, which restricts our ability to make causal inference. However, the inversion of cause and effect is unlikely to occur, because the onset of DM occurred long after birth, and the association between birth weight and DM is not established well enough to bias the participants' reports. Although it has been pointed out that adjustment for BMI may lead to a false association between birth weight and adult DM,^16^ the present study confirmed that the association remained in the analysis stratified by BMI. The use of self-reported data on DM status is another limitation. However, our sensitivity analysis using a dependent variable of DM defined using a combination of a fasting plasma glucose level and the use of DM drugs and an analysis limiting to DM diagnosed within the previous 3 years revealed the same results. In addition, a study that examined the validity of self-reported diabetes in a general Japanese population reported a sensitivity of 70.4% and a specificity of 97.3%.^17^ In general, limited sensitivity in disease classification is unlikely to bias relative risk estimates if specificity is very high.^18^ Because the women in the present study were healthcare workers, the accuracy of their responses is assumed to be higher than that for the general population.

The validity of self-reported birth weight data was examined in a subsample of participants from the present study. In 120 women who provided birth weight in both the baseline survey and the validity survey, the test-retest consistency of birth weight was high (kappa coefficient: 0.73, *p* < 0.001). For 24 women who provided written records (such as maternal and child health handbooks) in the validity survey, the consistency of birth weight categories between these records and the responses to the baseline survey was also high (kappa coefficient: 0.72, *p* < 0.001). These results show a sufficiently high accuracy of self-reported birth weight data in the female nurses included in this study.

In conclusion, birth weight and its percentile for gestational age were associated with adult-onset DM. Attention should be paid to the risk of DM among women born with low weight, even when their current BMI is normal.

## Funding

This work was supported by JSPS (Japan Society for the Promotion of Science) Grants-in-Aid for Scientific Research (24501367 and 18390195).

## Conflicts of interest

None declared.

## Author contribution

Kota Katanoda: Conception and design, analysis, interpretation of data, and drafting the article. Mitsuhiko Noda, Atsushi Goto, Hideki Mizunuma, Jung Su Lee: Interpretation of data, and revising the article. Kunihiko Hayashi: Conception and design, interpretation of data, and revising the article.
