# Supplementary material for: Impact of birth weight on adult-onset diabetes mellitus in relation to current body mass index: The Japan Nurses' Health Study
Source: J Epidemiol. 2017 Jun 20;27(9):428–34. doi: 10.1016/j.je.2016.08.016 (PMC5565756; doi:10.1016/j.je.2016.08.016)
Supplement: Supplementary file 1 [file mmc1.pdf]

eTable 1. Odds ratio for developing adult-onset DM per 100 g increase in birth weight by birth weight percentile category and parental DM history[a, b]

	Birth weight percentile category								
	Small for gestational age (< 10 percentile)			Appropriate (10–89 percentile)			Large for gestational age (90 percentile <=)		
(Number of participants; %)	1454 8.2%)			13,071 74.0%)			3144 17.8%)		
	OR	95% CI		OR	95% CI		OR	95% CI	
Overall	0.90	0.79	1.04	0.96	0.90	1.03	**0.91**	0.84	0.99
By history of paternal DM									
Yes	0.88	0.67	1.16	0.97	0.86	1.11	0.85	0.68	1.05
No	0.90	0.76	1.05	0.96	0.88	1.04	0.92	0.84	1.02
By history of maternal DM									
Yes	0.78	0.50	1.21	1.10	0.95	1.27	1.04	0.81	1.35
No	0.91	0.78	1.06	**0.92**	0.86	0.99	**0.88**	0.80	0.97

CI, confidence interval; DM, diabetes mellitus; OR, odds ratio.

[a] Birth weight percentile was calculated using the population average and standard deviation of birth weight according to gestational period. Participants with unknown gestational age (8701 women) or extreme birth weight percentile (703 women) were excluded.

[b] Adjusted for age (continuous)
